# Prevalence of chronic kidney disease in Tunisian diabetics: the TUN-CKDD survey

**DOI:** 10.1186/s12882-024-03501-5

**Published:** 2024-02-25

**Authors:** Jannet Labidi, Amel Harzallah, Badereddine Ben Kaab, Ikram Mami, Sahar Agrebi, Awatef Azzabi, Soumaya Chargui, Mayssa Hadj-Brahim, Mouna Hammouda, Saifeddine Azaiez, Syrine Tlili, Olfa Lajili, Hela Antit, Yosra Hasni, Sarra Chenik, Farhat Chelbi, Lamia Rais, Habib Skhiri

**Affiliations:** 1 Department of Nephrology, Military Hospital of Instruction of Tunis, Tunis, Tunisia; 2 Department of Nephrology, Charles Nicolle Hospital of Tunis, Tunis, Tunisia; 3 Department of Nephrology, Internal Security Force Hospital of La Marsa, Tunis, Tunisia; 4 Department of Nephrology, La Rabta Hospital of Tunis, Tunis, Tunisia; 5 Department of Nephrology, Sahloul Hospital of Sousse, Sousse, Tunisia; 6 Department of Nephrology, Tahar Sfar Hospital of Mahdia, Mahdia, Tunisia; 7 Department of Nephrology, Fattouma Bourguiba Hospital of Monastir, Monastir, Tunisia; 8 Private Sector, Ben Arous, Tunisia; 9 National Institute of Nutrition, Tunis, Tunisia; 10 Basic Care Center of Ezzahra, Ben Arous, Tunisia; 11 Department of Endocrinology, Farhat Hached Hospital of Sousse, Sousse, Tunisia; 12 Department of Cardiology, Military Hospital of Tunis, Tunis, Tunisia; 13 Department of Internal Medicine, Regional Hospital of Gafsa, Gafsa, Tunisia; 14 Tunisian Association of Nephrology, Dialysis, and Transplantation, Tunis, Tunisia

**Keywords:** Diabetes mellitus, Chronic kidney failure, Albuminuria, Prevalence

## Abstract

**Background:**

In Tunisia, the prevalence of diabetes mellitus increased from 15.5% on 2016 to 23% by 2023. While Chronic Kidney Disease (CKD) stills the most dreaded complications of diabetes, studies on the prevalence of chronic kidney disease non-dialysis diet are scarce. The aim of this study was to assess the prevalence of chronic kidney disease among the Tunisian diabetic population based on investigators’ specialty, demographic criteria (gender, age, duration of diabetes and geographic distribution) and diagnosis criteria (albuminuria and/or eGFR).

**Methods:**

This observational, multicentric, and cross-sectional study enrolled all diabetic subjects from all regions of Tunisia with at least 3 months of follow-up before the inclusion date, from 09 January to 08 February 2023. CKD diagnosis was established based on the KDIGO guidelines. The study was carried out at medical departments and ambulatory clinics of different healthcare providers. Baseline data were collected by investigators using an electronic case report form (eCRF). Continuous variables were described by means, median, standard deviation, and quartiles. Categorical data were tabulated in frequencies and percentages.

**Results:**

The overall prevalence of CKD among the 10,145 enrolled patients with diabetes mellitus was 38.7% with a 95%CI [37.8-39.6%]. 50.9% were male, with a mean age of 67.5 (± 11.3) years. The mean diabetes duration was 16.1 years (± 8.9). The highest CKD prevalence was noted among nephrologists (82.2%), while it was similar between the cardiologists and the primary care physicians (30.0%). CKD prevalence was highest among males (43.0% versus 35.1%) and increased proportionally with patients’ age and diabetes duration. CKD was more frequent in the Mid-East Area when compared to other regions (49.9% versus 25.3 to 40.1% in other regions). Albuminuria was present within 6.6% of subjects with CKD, and it was found an estimated glomerular filtration rate (eGFR) < 60 ml/min/1.73 m² within 13.3% of subjects wit h CKD. 18.9% had both criteria.

**Conclusions:**

In Tunisia, CKD among diabetics had a prevalence of 38.7%, approaching European prevalence. The prevalence discrepancy worldwide of CKD can be improved with a larger population size and by implementing standardized practices.

## Introduction

The International Diabetes Federation reports a continuous increase in diabetes prevalence reaffirming diabetes as a major global health concern. By 2021, 537 million adults aged between 20 and 79 years are living with diabetes around the world. This number is predicted to rise to 643 million by 2030 [1]. In Tunisia, the prevalence of diabetes mellitus reached 15.5% by 2016 [2]. On 2023, the ATERA survey estimated the prevalence of type 2 diabetes at 23% [3].

Chronic Kidney Disease (CKD) is one of the most dreaded complications of diabetes. A systematic review estimated its prevalence globally to be 57% [4, 5]. Besides, diabetes mellitus has emerged as the most prevalent cause of end-stage renal disease (ESRD) [6], cardiovascular events, and early death in developing and developed countries [4].

In Africa, in a recent systematic review, the prevalence of CKD was estimated to range between 2% and 41% while the prevalence of CKD among diabetic subjects ranged from 11 to 90%. In North Africa, CKD prevalence varies from 11 to 20% [8].

CKD problem among diabetic subjects remains underestimated on the entire continent due to a lack of epidemiological information from different African countries. In Tunisia, studies on the prevalence of chronic kidney disease non-dialysis diet are scarce.

**Our study aimed to assess the prevalence of chronic kidney disease among the Tunisian diabetic population based on investigators’ specialty, demographic criteria (gender, age, duration of diabetes and geographic distribution) and diagnosis criteria (albuminuria and/or eGFR).**

## Methods

### Study design and patients’ inclusion

This observational, multicentric, and cross-sectional study enrolled all diabetic subjects with at least 3 months of follow-up before the inclusion date, from 09/01/2023 to 08/02/2023.

Diabetes mellitus was defined as a fasting plasma glucose ≥ 7 mmol/L on two or more occasions, a random blood glucose ≥ 11.1 mmol/L with symptoms of hyperglycemia or hyperglycemic crisis, HbA1C > 6.5% [9] or patients under treatment of type 2 diabetes.

The inclusion criteria were as follow:

- Age ≥ 18 years old,
- Type 1 or Type 2 diabetes,
- Follow-up at the consultation for at least more than 3 months,
- Informant consent of the patient.

Subjects with chronic dialysis, kidney transplant, and pregnant women were non-included. When informed consent was withdrawn, diagnosis of chronic renal disease was not well established, and/or missing data, subjects were excluded from the study. The recruited individuals in this non-interventional study underwent clinical evaluations and get routine medical care as decided by their treating physicians. Participation was entirely voluntary.

### Chronic kidney disease diagnosis

In the current study, based on the Kidney Disease Improving Global Outcomes (KDIGO) guidelines [10], chronic kidney disease was defined by albuminuria and/or estimated glomerular filtration rates (eGFR) < 60 ml/min/1.73 m² for three months. Normative values for albuminuria are generally expressed as the urinary loss rate and referred to as Albumin Excretion Rate (AER) or Albumin-to-Creatinine Ratio (ACR). The threshold for AER is ≥ 30 mg/24 hours sustained for > 3 months to indicate CKD (which is equivalent to an ACR > 30 mg/g or > 3 mg/mmol). Albuminuria categories are detailed in Table 1.

**Table 1:** Categories for albuminuria [10]

	A1	A2	A3
AER, mg/24hours	< 30	30–300	> 300
PER, mg/24hours	< 150	150–500	> 500
ACR			
mg/mmol mg/g	< 3 < 30	3–30 30–300	> 30 > 300
PCR			
mg/mmol mg/g	< 15 < 150	15–50 150–500	> 50 > 500

AER: albumin excretion rate, PER: protein excretion rate, ACR: albumin-to-creatinine ratio, PCR: protein-to-creatinine ratio.

For each patient, estimated glomerular filtration rates were calculated based on The Modification of Diet in Renal Disease Study equation [11, 12]:

$$ \begin{array}{*{20}{c}}{{\rm{eGFR = 175}}\,{\rm{x}}\,{\rm{standardized}}\,{\rm{Serum - Creatinin}}{{\rm{e}}^{{\rm{ - 1}}{\rm{.154}}}}{\rm{x}}\,{\rm{ag}}{{\rm{e}}^{{\rm{ - 0}}{\rm{.203}}}}}\\{{\rm{x}}\,{\rm{1}}{\rm{.212}}\,\left( {if\,black} \right){\rm{x}}\,{\rm{0}}{\rm{.742}}\,\left( {if\,female} \right)}\end{array} $$

GFR categories are:

- **Category G1**: GFR ≥ 90 ml/min/1.73m^2^
- **Category G2**: GFR 60–89 ml/min/1.73m^2^
- **Category G3a**: GFR 45–59 ml/min/1.73m^2^
- **Category G3b**: GFR 30–44 ml/min/1.73m^2^
- **Category G4**: GFR 15–29 ml/min/1.73m^2^
- **Category G5**: GFR < 15 ml/min/1.73m^2^

### Sites and investigators selection

The study was carried out for one month at medical departments and ambulatory clinics of primary care physicians (general physicians and family medicine specialists), nephrologists, specialists in nutrition and metabolic diseases, internal medicine physicians, cardiologists, or other healthcare providers in charge of diabetic patients. Tunisia is divided into seven regions (Great Tunis, North East, North West, Mid-East, Mid-West, South East, and South West). For each region, a list of prospective sites was established, and all sites were invited to participate in the study.

### Data collection

Informed consent was obtained from all the study participants, and data were collected at baseline by investigators using an electronic case report form (eCRF) via a web-based data capture system managed by the DACIMA Clinical Suite®. The electronic data capture platform complies with the FDA 21 CFR part 11 requirements (Food and Drug Administration 21 Code of Federal Regulations part 11), the HIPAA specifications (Health Insurance Portability and Accountability Act), and the ICH standards (International Conference on Harmonization).

Baseline data included demographic and anthropometric data, presence of comorbidities, medical history of type 2 diabetes, and diagnostic tests for chronic renal failure.

### Statistical analysis

The data reviewing and data cleaning were performed through the electronic data capture system (Dacima). After the database lock, the statistical analysis was done to describe the population outcomes according to the study objectives. Continuous variables were described by means, median, standard deviation and quartiles. Categorical data were tabulated in frequencies and percentages. No inferential statistics were performed for this study. The 95% confidence interval was estimated for the primary outcome.

## Results

### Study design

In an observational multicentric cross-sectional study, 11,033 subjects were eligible for the national registry of Chronic Kidney Disease TUN-CKDD. 18 subjects did not give their informed consent and were non-included. 11,015 were enrolled and among them, 750 subjects were excluded for missing data.

**Fig. 1:**
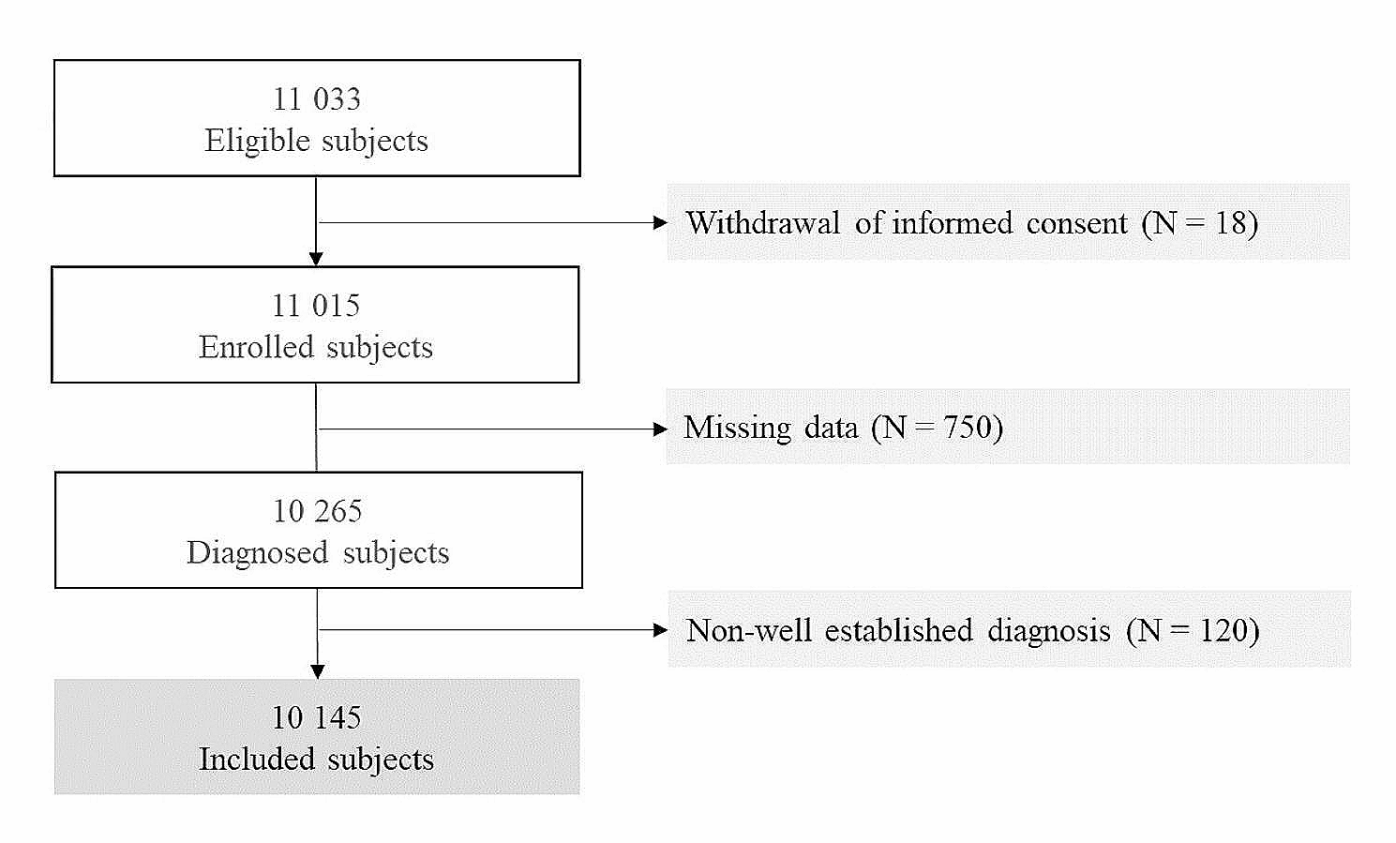
Flowchart of the participants

10 265 subjects underwent the diagnostic tests, and 120 subjects were excluded for a non-well-established diagnosis of CKD. The flowchart of the participants is presented in Fig. 1.

### Prevalence of chronic kidney disease

The overall prevalence of CKD in patients with diabetes mellitus was 38.7% with a 95%CI [37.8-39.6%].

Among the 10 145 subjects enrolled, 3929 had an eGFR < 60 ml/min/1.73 m² and/or a ACR ≥ 3 mg/mmol.

The cohort with CKD and diabetes mellitus was 50.9% male, with a mean age of 67.5 (± 11.3) years. The mean diabetes duration was 16.1 years (± 8.9) with a BMI of 28.6 kg/m^2^ and an HbA1c level of 8.2% (± 1.7). Sociodemographic and clinical data are presented in Table 2.

#### Prevalence based on investigators’ specialty

**Fig. 2:**
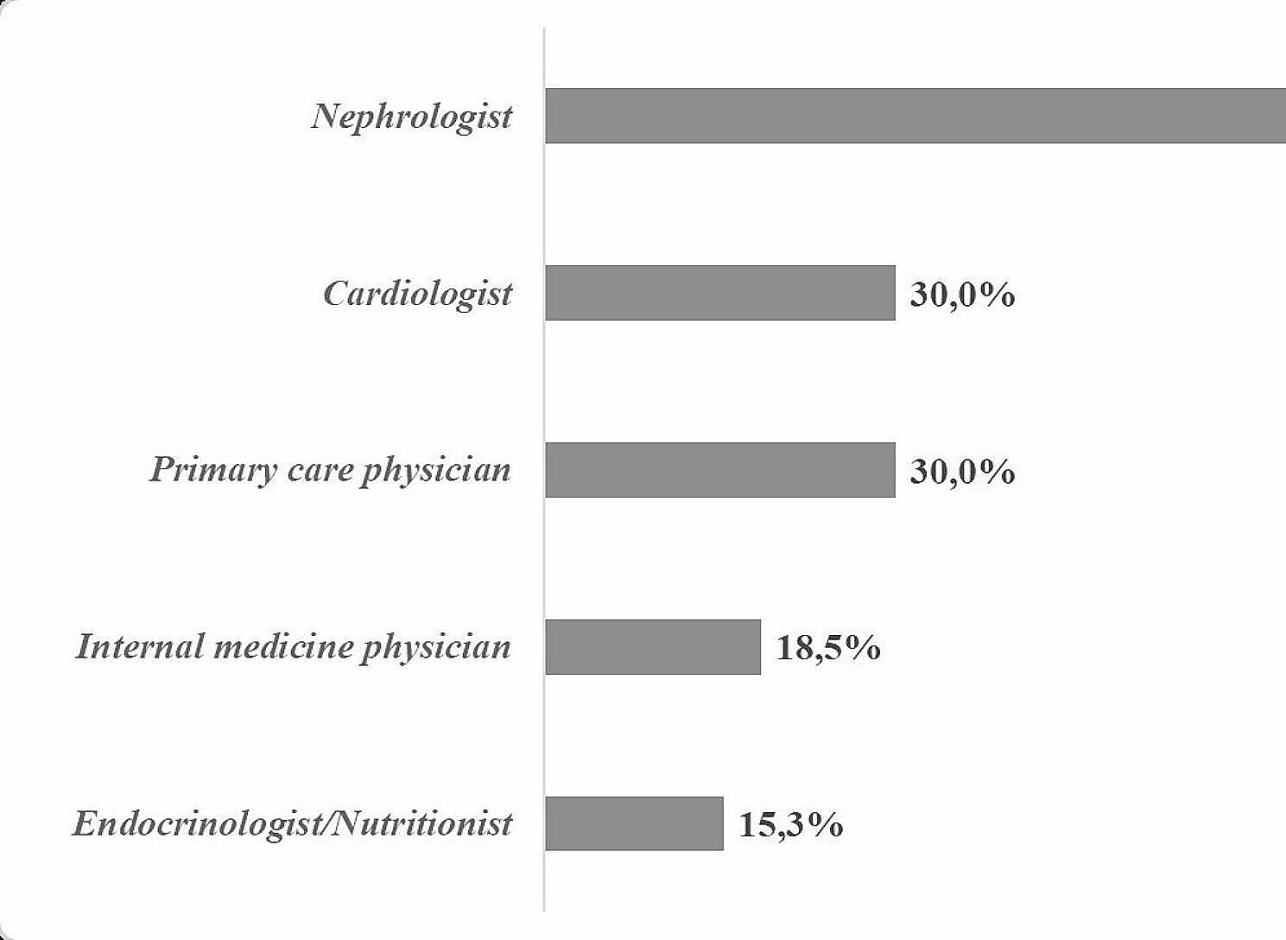
Chronic kidney disease prevalence based on investigators’ specialty

This national survey was conducted by a several groups of specialists. The highest CKD prevalence was noted among nephrologists (82.2%), while it was similar between the cardiologists and the primary care physicians (30.0%), Fig. 2.

Nephrologists reported the highest prevalence of CKD at 82.2%. This likely reflects their specific focus on kidney diseases, making them more likely to encounter and diagnose CKD patients.

Cardiologists and primary care physicians: Reported similar prevalence levels. This could be due to several factors, such as:Cardiologists: While not primarily focused on kidney health, they may encounter CKD patients due to its common co-occurrence with cardiovascular issues.Primary care physicians: As the first point of contact for many patients, they see a wide range of health conditions, including CKD.

**Table 2:** Prevalence of chronic kidney disease according to patients’ characteristics

	Overall population *N* = 10 145	Male *N* = 4655	Female *N* = 5490
Overall CKD prevalence	3929 (38.7)	2001 (43.0)	1928 (35.1)
Age, mean (± SD), year	67.5 ± 11.3	66.7 ± 11.4	68.4 ± 11.2
Diabetes mellitus			
Type 2, No (%) Type 1, No (%)	3757 (39.4) 172 (27.9)	1915 (44.0) 86 (28.5)	1842 (35.6) 86 (27.3)
Diabetes duration, mean (± SD), year	16.1 ± 8.9	16.1 ± 9.0	16.1 ± 8.9
Comorbidities			
Hypertension, No (%) Dyslipidemia, No (%) Hyperuricemia, No (%)	3199 (48.5)​ ​​1,893 (40.1)​ 363 (70.2)	1617 (54.5) 941 (44.9) 165 (69.6)	1582 (43.7) 952 (36.3) 198 (70.7)
Smoking			
Non-smoker, No (%) Active smoker, No (%) Weaned, No (%)	2858 (36.9) 575 (41.5) 496 (49.2)	969 (40.7) 546 (42.2) 486 (49.7)	1889 (35.2) 29 (32.2) 10 (33.3)
Clinical data			
eGFR mL/min/1.73 m^2^, median (± IQR) μalbuminuria mg/mmol, median (± IQR) BMI, mean (± SD), kg/m^2^ < 25, No (%) 25-29.9, No (%) 30-34.9, No (%) ≥ 35, No (%) HbA_1c_ (%), mean (± SD)	42.9 (29.9; 55.1) 750 (208.3; 1,975.3) 28.6 ± 5.6 946 (40.5) 1658 (38.6) 1202 (37.5) 123 (40.7) 8.2 ± 1.7	44.6 (30.8; 56.7) 940 (300; 2,000) 27.6 ± 5.5 608 (42.6) 912 (42.0) 451 (45.3) 30 (50.0) 8.2 ± 1.6	40.9 (28.9; 53.1) 600 (135.5; 1,653) 29.9 ± 5.5 338 (37.1) 746 (35.1 751 (34.0) 93 (38.4) 8.3 ± 1.8
Sedentary, N (%)	1834 (38.7)	787 (44.2)	1047 (36.2)
Educational level			
Unschooled, No (%) Primary school, No (%) Secondary school, No (%) University, No (%) Not specified, No (%)	758 (42.9) 991 (36.6) 693 (28.9) 231 (28.7) 1256 (51.0)	133 (44.2) 502 (44.5) 504 (33.9) 174 (33.4) 688 (56.5)	625 (42.6) 489 (30.9) 189 (20.8) 57 (20.1) 568 (45.5)

#### Prevalence of chronic kidney disease based on gender

Among males included in the study population, 43.0% had CKD versus 35.1% of females.

eGFR and ACR were higher among males with a median of 44.6 (30.8; 56.7) versus 40.9 (28.9; 53.1) mL/min/1.73 m^2^ and 940 (300; 2000) versus 600 (135.5; 1653) mg/mmol respectively (Table 2).

#### Prevalence of chronic kidney disease based on age and diabetes duration

**Fig. 3:**
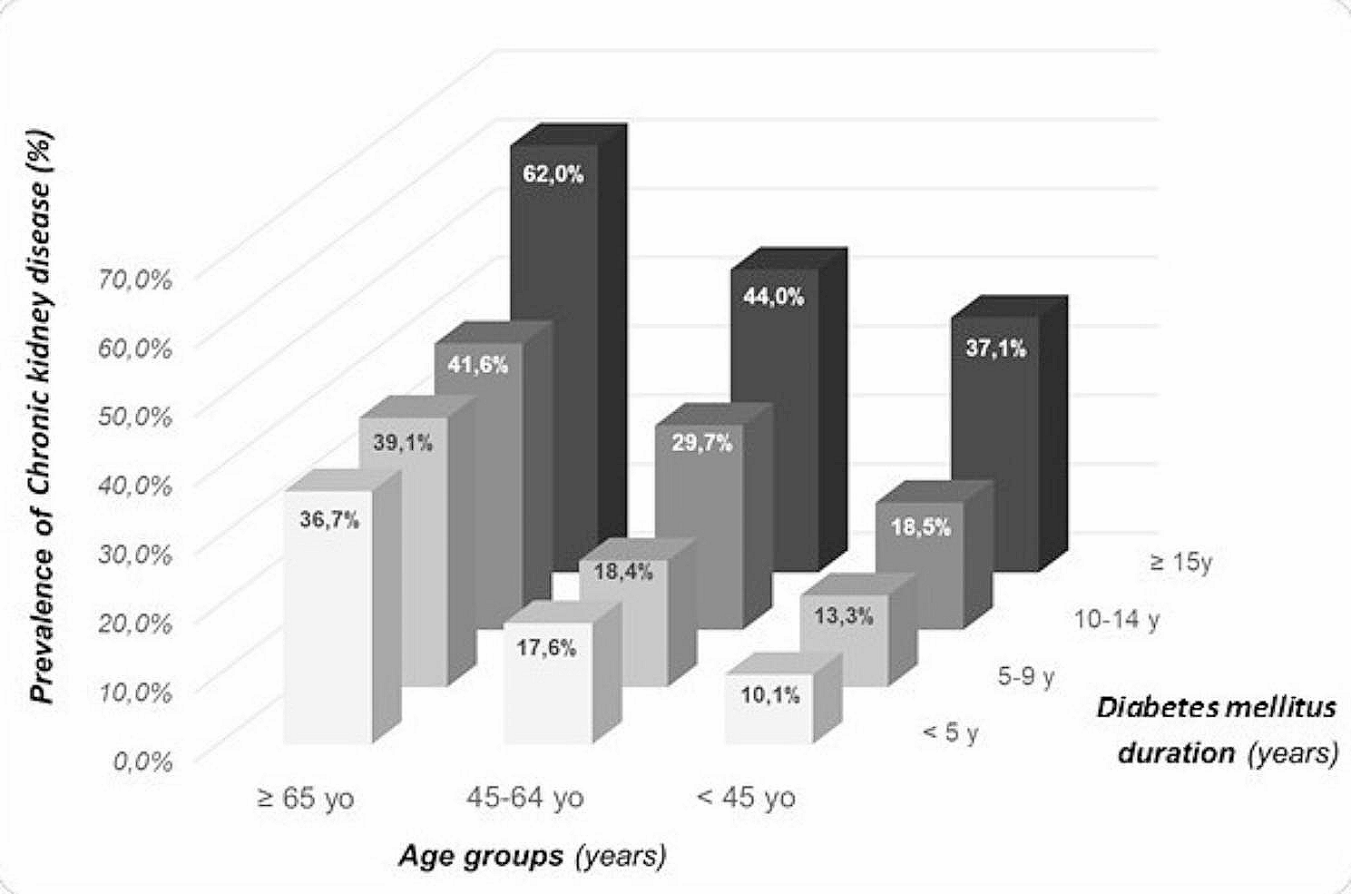
Three-dimensional representation of the prevalence of chronic kidney disease among diabetic subjects according to age and diabetes duration categories

Figure 3 shows the increase of CKD prevalence proportionally with patients’ age and diabetes duration. The lowest prevalence was in the below 40 years’ age group with diabetes duration < 5 years (10.1%), while the highest was with duration above 15 years in the age group over 65 years, amounting to 62.0%.

#### Prevalence of chronic kidney disease based on geographic distribution

**Fig. 4:**
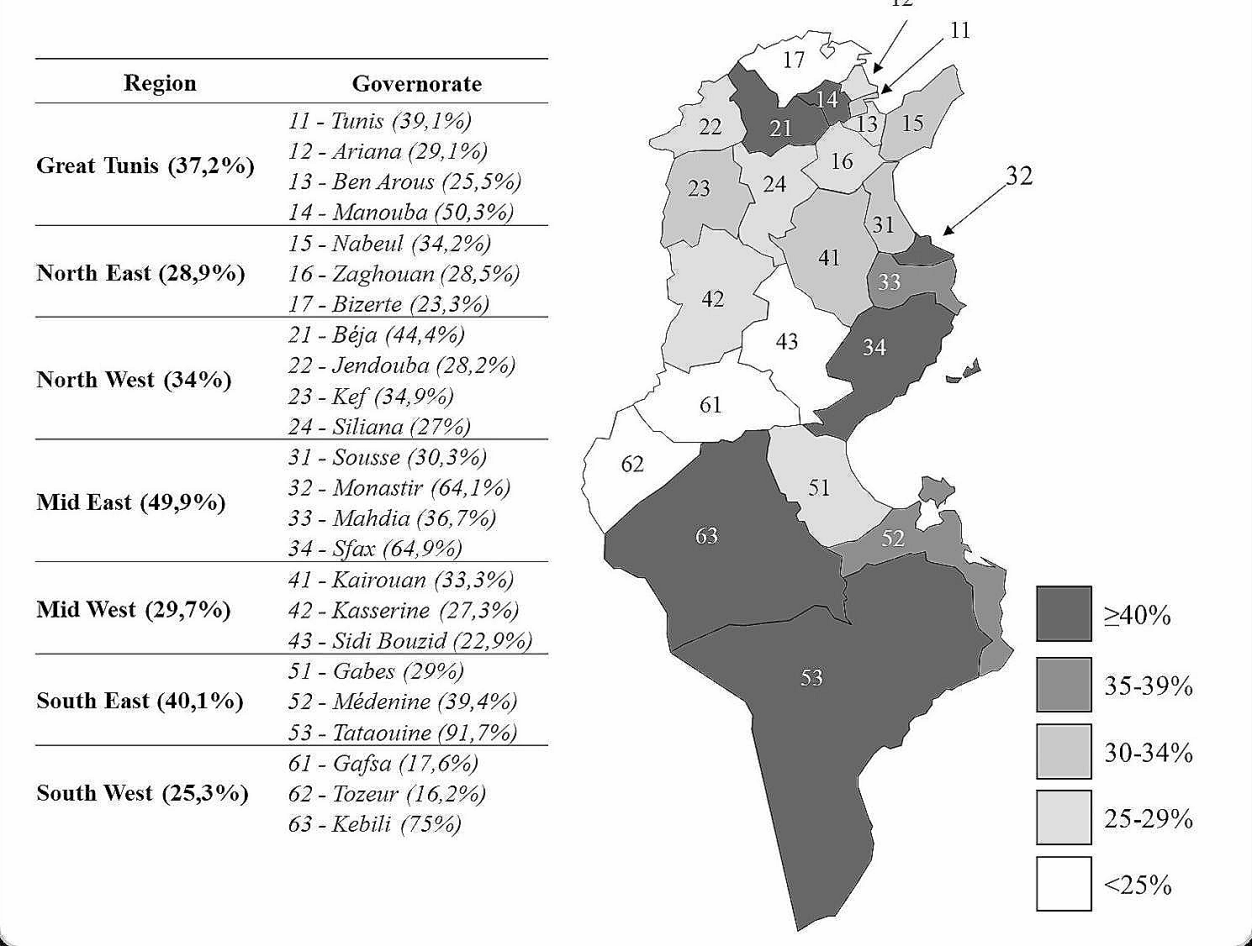
Chronic kidney disease prevalence based on geographic distribution

CKD prevalence was higher in the Mid-East Area when compared to other regions (49.9% versus 25.3 to 40.1% in other regions) (Fig. 4).

In the Greater Tunis Area, the prevalence of CKD varies between 25.5% (Ben Arous) and 50.3% (Manouba). The lowest prevalence was noted in Tozeur (16.2%).

#### Prevalence of chronic kidney disease based on diagnosis criteria

As KDIGO guidelines defined Chronic Kidney Disease as albuminuria and/or eGFR < 60 ml/min/1.73 m² [10], the survey determined the prevalence of each criterion.

**Fig. 5:**
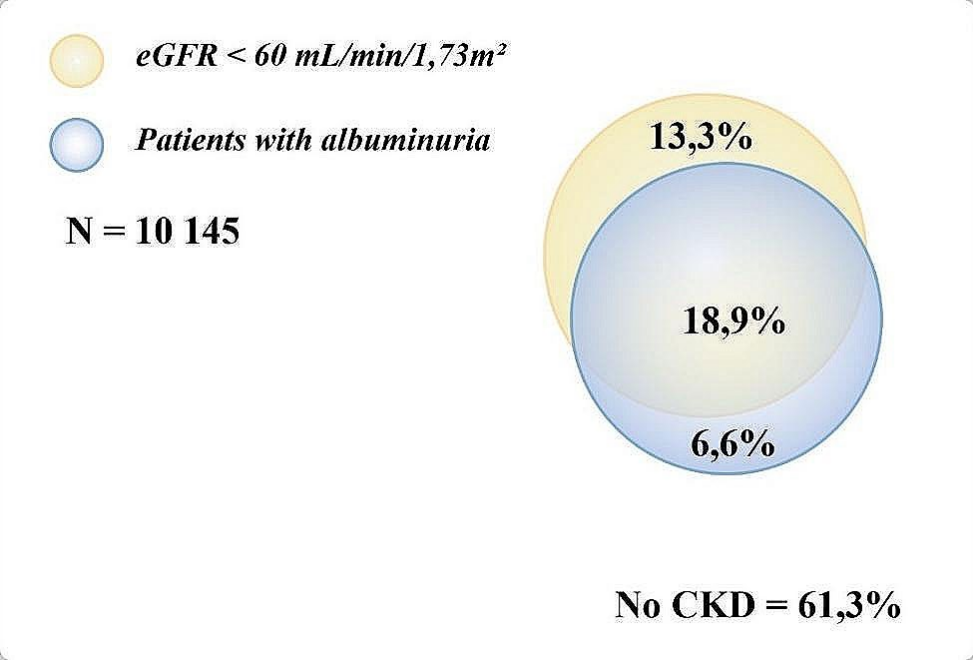
Distribution of patients with chronic kidney disease based on their clinical criteria

Albuminuria was present within 6.6% of subjects with CKD, and it was found an eGFR < 60 ml/min/1.73 m² within 13.3% of subjects with CKD. 18.9% had albuminuria and eGFR < 60 ml/min/1.73 m² (Fig. 5).

The prevalence of CKD based on GFR, and albuminuria categories is detailed in Table 3.

**Table 3:** Prevalence of chronic kidney disease based on albuminuria and estimated glomerular filtration rate categories

			Albuminuria categories No (%)				
		Albuminuria unmeasured		A1​	A2​	A3​	Total​
eGFR categories No (%)	**G1​**	-​		-​	174 (4.)​	69 (1.8)​	**243 (6.2)**​
	**G2​**	-​		-​	262 (6.7)​	161 (4.1)​	**423 (10.8)**​
	**G3a​**	260 (6.6)​		262 (6.7)​	311 (7.9%)​	291 (7.4)​	**1124 (28.6)**​
	**G3b​**	276 [7]​		182 (4.6)​	310 (7.9)​	377 (9.6)​	**1145 (29.1)**​
	**G4​**	205 (5.2)​		68 (1.7)​	144 (3.7)​	335 (8.5)​	**752 (19.1)**​
	**G5​**	77 [2]​		17 (0.4)​	27 (0.7)​	121 (3.1)​	**242 (6.2)**​
	**Total​**	**818 (20.8)**​		**529 (13.5)**​	**1228 (31.3)**​	**1354 (34.5)**​	**3929 (100**​

## Discussion

This prospective multicentric study provides insights into epidemiologic data of chronic kidney disease among diabetic subjects in the Tunisian population. To the best of our knowledge, the Tunisian Chronic Kidney Disease among Diabetics (TUN-CKDD) is the first national register for diabetic kidney disease. By 2023, the prevalence of CKD in Tunisian diabetic subjects was 38.7%. Among them, 6.6% exhibited albuminuria, 13.3% had an eGFR below 60 ml/min/1.73 m² and 18.9% fulfilled both criteria. The prevalence of CKD in T2D was 39.4% while it was 27.9% among T1D. Patients with T1D are typically diagnosed at a younger age being healthier and with fewer comorbidities compared to patients with T2D. These factors may contribute to the comparatively lower prevalence of CKD observed in this patient group [4]. The prevalence of CKD among patients with T2D varies across the world.

In Africa, it ranges from 11 to 90%. In North Africa, CKD prevalence varies from 11 to 20% [8], while in Sub-Saharan Africa, its prevalence seems to be higher ranging from 4 to 24% [13]. In the Middle East, The Saudi study was the largest registry including 54.670 Saudi type 2 diabetic patients. The prevalence of diabetic nephropathy was 10.8%, among them 14% had ESRD [14]. However, data on the prevalence of the earlier stages of CKD in the Middle East is still sparse.

In the United States, diabetes mellitus is the leading cause of kidney failure. By 2014, the prevalence of CKD among diabetics was estimated at 25% [15] and would attend 54% by 2030 [16]. In Canada, CKD prevalence among T2D reaches 48% [17]. In the European Union, the prevalence of CKD among diabetics varies among countries. The Finnish registry showed a prevalence of CKD among T2D subjects of 30.1% [18], while in the Greek registry, the overall prevalence of CKD among diabetics was 45% [19]. The prevalence of CKD in Tunisia seems to be near to that of European countries in contrast to the United States and Canada, where the prevalence of CKD was higher. In Asia, reported CKD prevalence among diabetics was lower than our study and ranged from 7% (95% CI 5.1–8.9%) in South Korea to 34.3% (95% CI 0.0–71%) in Singapore [20] and the Chinese registry showed a prevalence of 29.6% [21].

Many factors may contribute to the observed differences in CKD prevalence worldwide. Either those differences are possibly due to true differences in the prevalence of CKD or due to the heterogeneity of studies. Indeed, we can attribute this variability to the registries size, ethnicity, socioeconomic disadvantage, educational attainment, lower therapy goal fulfillment, patient therapeutic education, screening rates, inadequate management of early complications, dietary and lifestyle factors, smoking, obesity, and genetic background [4]. Moreover, factors beyond diagnostic criteria can influence the prevalence of kidney disease among individuals with diabetes.

Based on the KDIGO 2012 Clinical Practice Guideline, CKD diagnosis is established when eGFR declines to < 60 ml/min/1,73 m² with/without a persistently elevated urinary albumin excretion [10]. Most studies have used only eGFR to determine the presence of CKD and therefore report on the prevalence of CKD stages 3–5. By 2020, The DISCOVER study, a global 3-year prospective observational study enrolling T2D subjects from 35 countries, defined CKD among diabetics as eGFR < 60 mL/min/1.73 m^2^ (estimated with the CKD-EPI formula) and found a prevalence of 56.8% in the Eastern Mediterranean Countries including Tunisia [22]. As well, the Nepali study reported CKD as the reduction of eGFR less than 60mL/min estimated by the Cockcroft-Gault equation and therefore, only stages 3–5 were considered as the presence of CKD. The frequency of CKD among Nepali T2D was then estimated up to 86.6% [23]. Although, similarly to our study, other investigators have considered the combination of albuminuria and decreased eGFR, as seen in previous studies [14, 18, 19].

One should notice that eGFR assessment methods may contribute also to the variability of CKD prevalence. Studies have shown that The MDRD study equation had the less bias and the highest accuracy to estimate eGFR in diabetic cohort trials [24, 25], while the CKD-EPI equation have less bias than the MDRD Study equation at GFR > 60 ml/min/1.73 m^2^ [26, 27], whilst the Cockcroft-Gault formula should be used in screening declining renal function in subjects with normal serum creatinine such as diabetic subjects with CKD stage 1 or 2 [28].

These results highlight the need for multiple sources of data from various countries to estimate and track CKD prevalence with standardized methods.

This nationwide registry involved physicians from different medical specialties, with primary care physicians being the most commonly involved. Nephrologists diagnosed the highest proportion of CKD prevalence among the diabetic subjects included in this study (82.2%), while primary care physicians and cardiologists accounted for 30% of CKD diagnoses and endocrinologists for only 15.3%. In Tunisia, the nephrologist delivers most CKD care. Like many chronic conditions, primary care physicians typically play a key role in identifying the presence of CKD in patients. Subsequently, they refer the patients to nephrologists for further evaluation and management. The KDIGO guidelines recommend referring patients to nephrologists when eGFR decreases below 30 ml/min/1.73 m² with/without increasing albuminuria [10]. Gender-related disparities have been observed in non-diabetic CKD. In many regions (excluding Japan, Singapore, and Thailand), the prevalence of CKD was higher among women compared to men. Notably, in France, Thailand, Portugal, and Turkey, CKD was twice as high in females [29].

Thereafter, several studies have explored sex differences in the prevalence of Diabetic Kidney Disease. Recent data suggest that diabetic men are at greater risk of developing CKD [30]. Indeed, our registry showed a higher prevalence of CKD among males (43%) when compared to females (35.1%). It has been shown that kidney function declines faster in men than women, possibly due to the renoprotective role of estrogens and progesterone [31] and to unhealthier lifestyles in men or the damaging effects of testosterone [32].

Furthermore, when considering specific CKD phenotypes, it is observed that diabetic men have a higher susceptibility to developing albuminuria, whereas women have a higher susceptibility to eGFR impairment and the development of ESRD [31].

It is important to acknowledge that eGFR formulas incorporate sex-specific corrections to account for disparities between men and women, such as the higher muscle mass typically found in men, resulting in higher creatinine levels and consequently lower eGFR values among women. Indeed, in our study, the median eGFR was slightly lower in women when compared to men (40.9 versus 44.6 mL/min/1.73 m^2^ respectively). The reasons behind these reported gender disparities in CKD are still largely unknown, but hormonal and genetic differences might be incriminated. Indeed, genetic susceptibility to CKD can be attributed to some identified genes and single nucleotide polymorphisms (SNPs) [33, 34]. Moreover, the involvement of epigenetic mechanisms along with their potential interactions with personal or environmental factors including gender, may also exert a significant influence [35, 36]. In our study, the prevalence of CKD among diabetic subjects increased proportionally with age and diabetes duration, as seen in other published data [14, 37, 38]. We report an accumulative effect of both age and duration on the prevalence of CKD, where it increases by twofold in patients with diabetes duration above 15 years, regardless of the patient’s age.

Moreover, in our study, the highest prevalence of CKD regardless of diabetes duration was among adults aged ≥ 65 years, as shown previously [14, 39]. According to the UK Prospective Diabetes Study, after a median follow-up of 15 years of diabetic subjects, approximately 28% would exhibit a decline in eGFR below 60 mL/min/1.73 m² along with albuminuria [40].

In Tunisia, in a large cohort study conducted from 2006 to 2008, the prevalence of CKD among diabetic subjects was 19.1% [41]. In our study, CKD prevalence was up to 38.7%. To the best of our knowledge, this is the first study to report the prevalence of CKD in the different regions of Tunisia. The highest prevalence was noted in the Mid-East Area (49.9% versus 25.3 to 40.1% in other regions) while the lowest was observed within the South West region (25.3%). This variability across regions in the Tunisian population might be explained by dietary and lifestyle factors or even genetic background since the different Tunisian regions are known to exhibit significant genetic diversity [42].

In our study, early stages of CKD were present among respectively 6.2% (Stage 1) and 10.8% (Stage 2) of diabetic subjects. Stages 3a and 3b exhibited higher prevalence (28.6% and 29.1%, respectively), followed by stage 4 (19.1%), while stage 5 was less common (6.2%).

In the Finnish study, early stages of CKD among diabetics were more frequent since 28.3% of patients had G1, 53.1% had G2 and G3a and G3b had a prevalence of 12.4% and 4.7% respectively [18]. As well, the Greek registry showed a higher prevalence of mild CKD [19].

Most patients with CKD, even stage 4, do not progress to the advanced stages of the disease, as mortality occurs before reaching ESRD [43–45].

Dalrymple et al. found that older persons with CKD were 13-fold more likely to die from any cause than to progress to ESRD [46].

Our study results should be interpreted with the consideration of a few limitations. Data analysis and results interpretation did not take into account the low recruiting potential of some sites that can prevent reaching optimal representativeness. Registry limitations include the possibility of incomplete data collection of medical history and for some patients’ albuminuria was not estimated.

The strengths of our study include its extensive scope that involved patients from across the entire national territory, encompassing both public and private healthcare systems in Tunisia. Moreover, the study was conducted by a diverse group of specialists, including primary care physicians, endocrinologists, nephrologists, and professionals from various other medical disciplines.

Prevalence of CKD among Diabetic Patients in Tunisia: Differences According to Gender and Age (BMC Nephrology, 2020): This study, while not focused on investigators’ specialty, analyzes CKD prevalence based on gender and age in the Tunisian diabetic population. It reveals higher prevalence among males and with increasing age, similar to your findings. Sex hormones and progression of chronic kidney disease in diabetic patients: (Kidney International, 2011): This review article discusses the complex interplay of sex hormones (estrogen, testosterone) and their impact on renal function and CKD progression, potentially explaining some gender differences.

In conclusion, this is the first study that carefully characterizes CKD prevalence across Tunisia. Diabetic kidney disease had a prevalence of 38.7%, approaching European prevalence. The prevalence discrepancy worldwide of CKD among diabetics can be improved with a larger population size and by implementing standardized practice. Either these differences are possibly due to true differences in the prevalence of CKD as well as to heterogeneity of the laboratory and sample selection methods, the effect of this variability about human and environmental factors, public health policies, and genetics on CKD prevalence needs further investigation. Epidemiological data allow us to better understand the Tunisian diabetic population with MRC. This will help us to assess the effectiveness of preventive measures and research to develop to improve the prognosis of these patients.

## Data Availability

The datasets generated and analyzed during the current study are not publicly available due to ethical concerns but are available from the corresponding author on reasonable request.
